# Love and Law

**Published:** 1894-05

**Authors:** 


LOVE AND LAW.—In one of his little couplets the immortal author of "Tam o'Shanter" announced his intention of catching a girl around her waist and kissing her until she blushed, or words to that effect. If Robert Burns lived in this country and in this generation, it would be well for him to employ counsel and take legal advice before engaging in any such hazardous business. Lawsuits are constantly arising from kissing. One girl will sue a kisser for breach of promise, another for assault and battery. All this is wrong. But perhaps the last kissing suit (but recently reported in the newspapers) is more justifiable, as it imparts to young men a wholesome lesson. Henry Ives, a young farmer in Bergen county, N. J., according to the story, wooed Miss Annie Rafferty, a comely country maiden of Manchester township, in the same State of mosquitoes and railroad commuters. The two were betrothed.

But ere long the course of true love was violently interrupted by a kiss. The details of the kiss have not as yet been satisfactorily given, but they doubtless will be when the case comes up for trial. At present it is uncertain whether Henry's teeth got tangled up in the gold filling of Annie's teeth, or whether there was a strong suction about his manner of kissing. At all events, it seems to be established, as alleged in the plantiff's declaration, that a gold filling came out and that the casuality was due to the kiss. Annie hinted that she would be subjected to some expense by reason of it and that it would be the proper thing for him to pay for replugging the tooth. The young man's apprehension seems to have been so dull as his kiss was emphatic and he failed to take the hint. A coolness ensued, and Annie, acting upon the advice of counsel, had the cavity plugged and sent the bill to Henry. This was Henry's opportunity. He should have paid the bill and, like John Gilpin, been overjoyed that though his betrothed was bent on pleasure, she had a frugal mind. But Henry missed the chance and the engagement is broken. Instead of the suit for Annie's hand he is now in a lawsuit. He refused to pay the bill and a writ was issued.

But Henry's first mistake was in his manner of kissing. Kisssing, according to the standard authorities, should be done gently. One of the late Lord Tennyson's girls tells us how "he drew her whole soul through her lips in one long, sweet caress." This kiss was probably very prolonged and apparently with some suction. But it was not violent. It takes less force to draw a girl's soul through her lips than to draw the plugs from her teeth. Tennyson's girl liked the kiss she got. Annie did not like hers. This is a pointer for young men. Kisses may be prolonged and lingering, but a girl can be won by a gentle kiss just as efficiently as by kissing every tooth out of her head. A woman is a gentle creature and requires gentle treatment. In one of the impulsive towns of the West some time ago an energetic young Western man went to hug the beautiful damsel who was to be his wife, and in his enthusiasm broke three of her ribs. The girl's father took the young man in his library and admonished him of the nature of woman and that tender sentiments could be expressed to a girl as effectually by a gentle pressure of the hand as by running her through a stone-crusher. Henry needs some such admonition as this.

The communication and exchange of microbes in kissing is another branch of the subject, but it is foreign to our present purpose.—ED. *Baltimore Sun.*

---

### Monthly Summary.

